# Correction: Torres et al. Twentieth-Century Paleoproteomics: Lessons from Venta Micena Fossils. *Biology* 2022, *11*, 1184

**DOI:** 10.3390/biology11101515

**Published:** 2022-10-17

**Authors:** Jesús M. Torres, Concepción Borja, Luis Gibert, Francesc Ribot, Enrique G. Olivares

**Affiliations:** 1 Departamento de Bioquímica y Biología Molecular III e Inmunología, Universidad de Granada, 18016 Granada, Spain; 2 Departament de Geoquímica, Petrologia i Prospecció Geològica, Universitat de Barcelona, 08028 Barcelona, Spain; 3 Museo de Prehistoria y Paleontología Josep Gibert, 18858 Orce, Spain; 4 Instituto de Biopatología y Medicina Regenerativa, Centro de Investigación Biomédica, Universidad de Granada, 18100 Armilla, Spain; 5 Unidad de Gestión Clínica Laboratorios, Hospital Clínico Universitario San Cecilio, 18016 Granada, Spain

There was an error in the original publication [1]. A correction has been made to add the **Acknowledgments Section**:

**Acknowledgments:** We thank K. Shashok for editing the use of English in this manuscript. 

The authors state that the scientific conclusions are unaffected. This correction was approved by the Academic Editor. The original publication has also been updated.
